# Effect of tumor suppressor gene cyclin-dependent kinase inhibitor 2A wild-type and A148T mutant on the cell cycle of human ovarian cancer cells

**DOI:** 10.3892/ol.2014.1867

**Published:** 2014-02-11

**Authors:** ZEHUA BIAN, YANG YU, TERIGELE YANG, CHAO QUAN, WENJING SUN, SONGBIN FU

**Affiliations:** 1 Laboratory of Medical Genetics, Harbin Medical University, Heilongjiang Higher Education Institutions, Harbin, Heilongjiang 150081, P.R. China; 2 Key Laboratory of Medical Genetics (Harbin Medical University), Heilongjiang Higher Education Institutions, Harbin, Heilongjiang 150081, P.R. China

**Keywords:** cyclin-dependent kinase inhibitor 2A, cyclin-dependent kinase inhibitor 2A-A148T, recombinant DNA technology, inducible expression

## Abstract

Single-base substitution may affect the function of genes. This study identified a single-base substitution of G for A in codon 148 of cyclin-dependent kinase inhibitor 2A (CDKN2A/p16) by sequencing human ovarian cancer cell line UACC-1598. As a tumor suppressor gene, the expression of CDKN2A/p16 should be strictly controlled. In order to control CDKN2A/p16 gene expression, an inducible pTUNE vector system was selected. Using recombinant DNA technology, a CDKN2A/p16-A148T and CDKN2A/p16-wild-type gene expression system was successfully constructed to investigate whether this single-base substitution affects the function of CDKN2A/p16. For the wild-type and the mutant, expression of CDKN2A/p16-green fluorescent protein fusion protein increased markedly following isopropyl-β-D-thiogalactoside induction, and was accompanied by significant G1 arrest in the transfected human ovarian cancer SKOV3 cell line. The inducible vectors used in this study, CDKN2A/p16-wild-type and CDKN2A/p16-A148T open reading frame, may be useful for further investigation into whether this somatic mutation could alter the function of CDKN2A/p16 as a tumor suppressor gene. In summary, CDKN2A/p16-A148T was identified in ovarian cancer cells, and this single-base substitution did not affect the ability of CDKN2A/p16 to arrest the cell cycle.

## Introduction

As a tumor suppressor gene regulating the cell cycle, cyclin-dependent kinase inhibitor 2A (CDKN2A/p16), is often found to be mutational in various types of human cancer (1). Loss of CDKN2A/p16 function arises by two main mechanisms: Homozygous deletion and 5′ CpG island hypermethylation with low transcription (2). Mutations in primary ovarian cancer and metastatic ovarian cancer do not occur at high frequency (3). However, the present study identified a single-base substitution from G to A in codon 148 of CDKN2A/p16 by sequencing human ovarian cancer cell line UACC-1598. The mutation results in the amino acid alanine (A) being substituted with threonine (T). It is known that the abnormal expression of cell cycle-regulating genes plays important roles in cancer cells (4), prompting the use of recombinant expression systems to investigate their function (5,6). The pTUNE vector establishes a gene expression system induced by isopropyl-β-D-thiogalactoside (IPTG) which utilizes repressor proteins and RNA interference to switch genes on or off while preserving gene function (7). Therefore, the present study used a pTUNE vector to construct human CDKN2A/p16-A148T mutant and CDKN2A/p16-wild-type gene expression systems in order to investigate the effect of CDKN2A/p16-A148T on the cell cycle.

## Materials and methods

### Cell culture and transfection

Human ovarian cancer cell line UACC-1598 was a gift from Dr Xin-Yuan Guan (University of Hong Kong, Hong Kong SAR, China). Human ovarian cancer cell line SKOV3 was purchased from American Type Culture Collection (Manassas, VA, USA). These cells were cultured in RPMI-1640 (Gibco, Carlsbad, CA, USA) containing 10% fetal bovine serum (PAA Laboraties GmbH, Pasching, Austria) at 37°C in a 5% CO_2_ humidified atmosphere.

Cells were seeded in six-well plates at a density of 5×10^6^ cells/well, and the constructed expression vectors were transfected into cells using Lipofectamine 2000 (Invitrogen Life Technologies, Carlsbad, CA, USA).

### Vector construction

The pTUNE inducible vector was obtained from OriGene Technologies Inc. (Rockville, MD, USA) as control vector. The primers specific for amplifying CDKN2A/p16 open reading frame (ORF) were synthesized by Invitrogen Life Technologies as follows: Primer 1 (hCDKN2A-SgfI), 5′-GAGGCGATGAGGCGATC GCATGGAGCCGGCGGCG-3′; primer 2 (hCDKN2A-XhoI), 5′-GCGCTCGAGATCGGGGATGTCTGAGGGACCTTC-3′; and primer 3 (hCDKN2A-XhoI), 5′-GCGCTCGAGAT CGGGGATGTCTGAGGGACCTTCCGCGGCATCTAG-3′.

The CDKN2A/p16-A148T ORF fragment was cloned from the cDNA template of UACC-1598 cells by polymerase chain reaction (PCR) with primers 1 and 2 specific to CDKN2A/p16. The PCR product was electrophoresed on a 2% agarose gel (Gene Tech Company Limited, Shanghai, China)and the predicted 490 bp fragment was extracted. The amplified ORF and the pTUNE inducible vector (OriGene Technologies, Inc.) were digested with endonuclease *XhoI* (New England Biolabs, Inc., Beijing, China) and *SgfI* (Promega Corporation, Madison, WI, USA). A ligation reaction was set up with linearized pTUNE vector and CDKN2A/p16 ORF using T4 DNA ligase (TransGen Biotech Co., Ltd., Beijing, China). The positive clone was picked up and the authenticity of the plasmid was confirmed by sequencing.

The CDKN2A/p16-wild-type ORF fragment was amplifed with primers 1 and 3 using the pTUNE-CDKN2A/p16-A148T vector template, which covers the mutation site and contains the wild-type base G substitution for correcting the sequence. The PCR product was separated by electrophoresis and the 490 bp fragment was extracted. The CDKN2A/p16-wild-type ORF was subsequently digested by the restriction enzymes *XhoI* and *SgfI* to construct the pTUNE-CDKN2A/p16-wild-type inducible vector. The positive clone was also sequenced for verification.

### Flow cytometric analysis of the cell cycle

Cells were harvested by trypsinization and fixed with 70% ethanol at 4°C for 24 h. The cells were then centrifuged at 500 × g for 5 min, washed twice with phosphate-buffered saline and stained with buffers provided in the Cycletest™ Plus DNA Reagent kit (BD Biosciences, Franklin Lakes, NJ, USA) for analysis by flow cytometry.

## Results

### Verification of the transfection efficiency of pTUNE-CDKN2A/p16-wild-type and pTUNE-CDKN2A/p16-A148T mutant vectors

**Figure 1:**
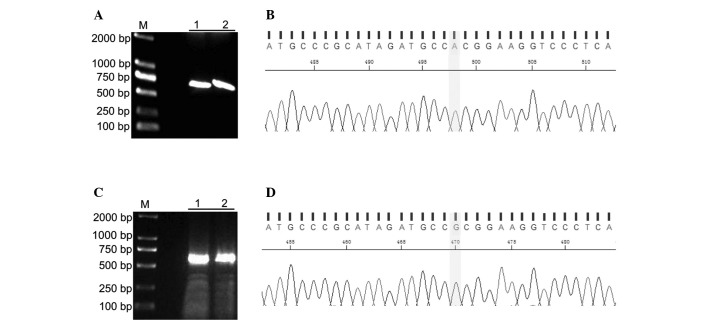
Construction of pTUNE-CDKN2A/p16-A148T and pTUNE-CDKN2A/p16-wild-type vectors. (A) Amplification of CDKN2A/p16-A148T ORF by PCR. Lanes 1 and 2 show the PCR products of the amplified CDKN2A/p16-A148T ORF fragments. (B) Verification of the pTUNE-CDKN2A/p16-A148T vector by DNA sequencing. The shadow indicates the presence of a single-base substitution. (C) Amplification of CDKN2A/p16-wild-type fragment by PCR. Lanes 1 and 2 show the PCR products of the amplified CDKN2A/p16-wild-type ORF fragment. (D) Verification of the pTUNE-CDKN2A/p16-wild-type vector by DNA sequencing. The shadow shows the original base in the wild-type ORF of CDKN2A. CDKN2A, cyclin-dependent kinase inhibitor 2A; ORF, open reading frame; PCR, polymerase chain reaction.

The present study identified a single-base substitution from G to A in codon 148 of CDKN2A/p16 by sequencing the human ovarian cancer cell line UACC-1598. This mutation resulted in the amino acid substitution of alanine (A) with threonine (T). pTUNE-CDKN2A/p16-A148T and pTUNE-CDKN2A/p16-wild-type expression vectors were successfully constructed (Figs. 1).

**Figure 2:**
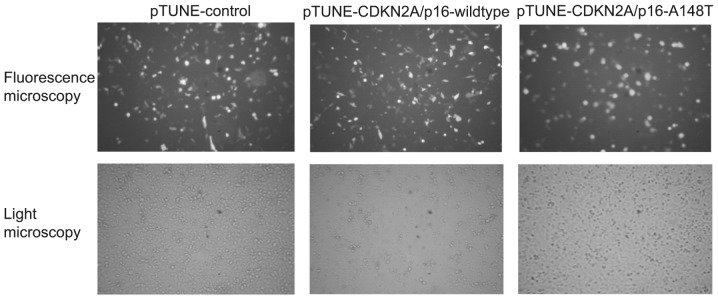
Expression of pTUNE-CDKN2A/p16 plasmids transiently transfected into SKOV3 cells and induced by IPTG. SKOV3 cells were transfected with pTUNE-control, pTUNE-CDKN2A/p16-wild-type or pTUNE-CDKN2A/p16-A148T plasmids for 24 h, followed by IPTG induction for 36 h. Magnification, ×100. CDKN2A, cyclin-dependent kinase inhibitor 2A; IPTG, isopropyl-β-D-thiogalactoside.

To verify the effect of the pTUNE-CDKN2A/p16-A148T and pTUNE-CDKN2A/p16-wild-type vectors, a CDKN2A homozygous deletion human ovarian cancer cell line, SKOV3, was selected (8). The constructed expression vectors pTUNE-CDKN2A/p16-A148T and pTUNE-CDKN2A/p16-wild-type were transfected into SKOV3 cells using Lipofectamine 2000 when the cells were ~80% confluent. After 24 h had elapsed, the cells were induced with 500 mM IPTG for an additional 36 h. As pTUNE-vectors contain green fluorescent protein (GFP) fragments, the efficiency of transfection was detected by fluorescence microscopy. The expression of wild-type and mutant CDKN2A/p16-GFP fusion protein increased markedly in the transfected SKOV3 cells following IPTG induction (Fig. 2). These results indicate that the vectors pTUNE-CDKN2A/p16-A148T and pTUNE-CDKN2A/p16-wild-type are effectively induced by IPTG and could be used for further study.

### Influence of CDKN2A/p16-A148T on the cell cycle

**Figure 3:**
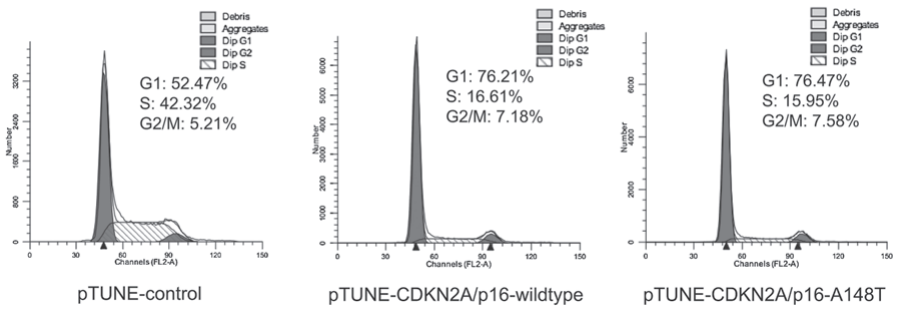
Cell cycle distribution of SKOV3 cells transiently transfected with pTUNE-CDKN2A/p16 and induced by IPTG. SKOV3 cells were transfected with pTUNE-control, pTUNE-CDKN2A/p16-wild-type or pTUNE-CDKN2A/p16-A148T plasmids for 24 h, followed by IPTG induction for 36 h. CDKN2A, cyclin-dependent kinase inhibitor 2A; IPTG, isopropyl-β-D-thiogalactoside.

CDKN2A/p16 is a cell cycle-regulating gene. In the present study, the effects of pTUNE-CDKN2A/p16-A148T and pTUNE-CDKN2A/p16-wild-type vectors on cell cycle regulation were examined. Cell cycle distribution of the transfected and IPTG-induced SKOV3 cells were determined by flow cytometry in three independent experiments. The results demonstrated that wild-type and A148T mutant expression of CDKN2A/p16 can induce cell cycle arrest in G1 phase (Fig. 3). This suggests that the amino acid substitution of A with T at codon 148 of CDKN2A/p16 does not influence the function of CDKN2A/p16 on cell cycle arrest.

## Discussion

CDKN2A/p16 is an important factor in cell cycle regulation (9). As a tumor suppressor gene, the expression of CDKN2A/p16 is strictly controlled. When overexpressed, location-specific CDKN2A/p16 expression is lost and it appears in the cytoplasm and the nucleus. This exerts abnormal influence on cell growth and division, resulting in cellular senescence and apoptosis (10). In order to control CDKN2A/p16 gene expression, an inducible pTUNE vector system was selected. This system is designed for IPTG-inducible expression, compared with a standard system in which IPTG treatment would induce only modestly elevated expression of proteins. In the present study, SKOV3 cells transfected with pTUNE-CDKN2A/p16-wild-type and pTUNE-CDKN2A/p16-A148T plasmids demonstrated significant G1 arrest compared with control cells. This illustrates that the amino acid substitution of A with T at CDKN2A/p16 codon 148 does not interfere with its role in cell cycle regulation. Wolf *et al* have previously demonstrated G1 phase arrest when CDKN2A/p16 was overexpressed in SKOV3 cells, which caused inhibition of cell growth (11). It has been confirmed that the CDKN2A/p16-A148T mutant exists in several types of cancer, including ovarian cancer, and, with the exception of certain populations, it is associated with cancer risk (3,12–14). However, the CDKN2A/p16-A148T mutant in melanoma does not exhibit impaired CDK4 binding function (15). By sequencing human ovarian cancer cell line UACC-1598, the present study also identified the CDKN2A/p16-A148T mutant. Assessment of the effect of the CDKN2A mutant on the cell cycle yielded results consistent with previous studies in which the CDKN2A mutant did not decrease the inhibitory activity of cyclin D1/CDK4 (15,16).

In conclusion, this study identified CDKN2A/p16-A148T mutation in ovarian cancer cells. The inducible expression vectors of CDKN2A/p16-wild-type and CDKN2A/p16-A148T utilized in the study could be useful for further investigation into whether this somatic mutation can alter the tumor suppression functions of CDKN2A/p16.
